# Supplementary material for: DNA methylation at enhancers identifies distinct breast cancer lineages
Source: Nat Commun. 2017 Nov 9;8:1379. doi: 10.1038/s41467-017-00510-x (PMC5680222; doi:10.1038/s41467-017-00510-x)
Supplement: Supplementary file 1 — Supplementary Information [file 41467_2017_510_MOESM1_ESM.pdf]

File Name: Supplementary Information
Description: Supplementary Figures and Supplementary Tables

File Name: Supplementary Data 1
Description: Enrichment of emQTL, Cluster 1 and Cluster 2 CpGs over the expected frequency of CpGs from the Illumina HumanMethylation450 across 111 ChiP-seq data sets from the MCF7 cell line.

File Name: Supplementary Data 2
Description: Genomic location of the CpGs in Cluster 2 and their annotation of CpGs from the Illumina HumanMethylation450k across 111 ChiP-seq data sets from the MCF7 cell line.

File Name: Peer Review File
Description:

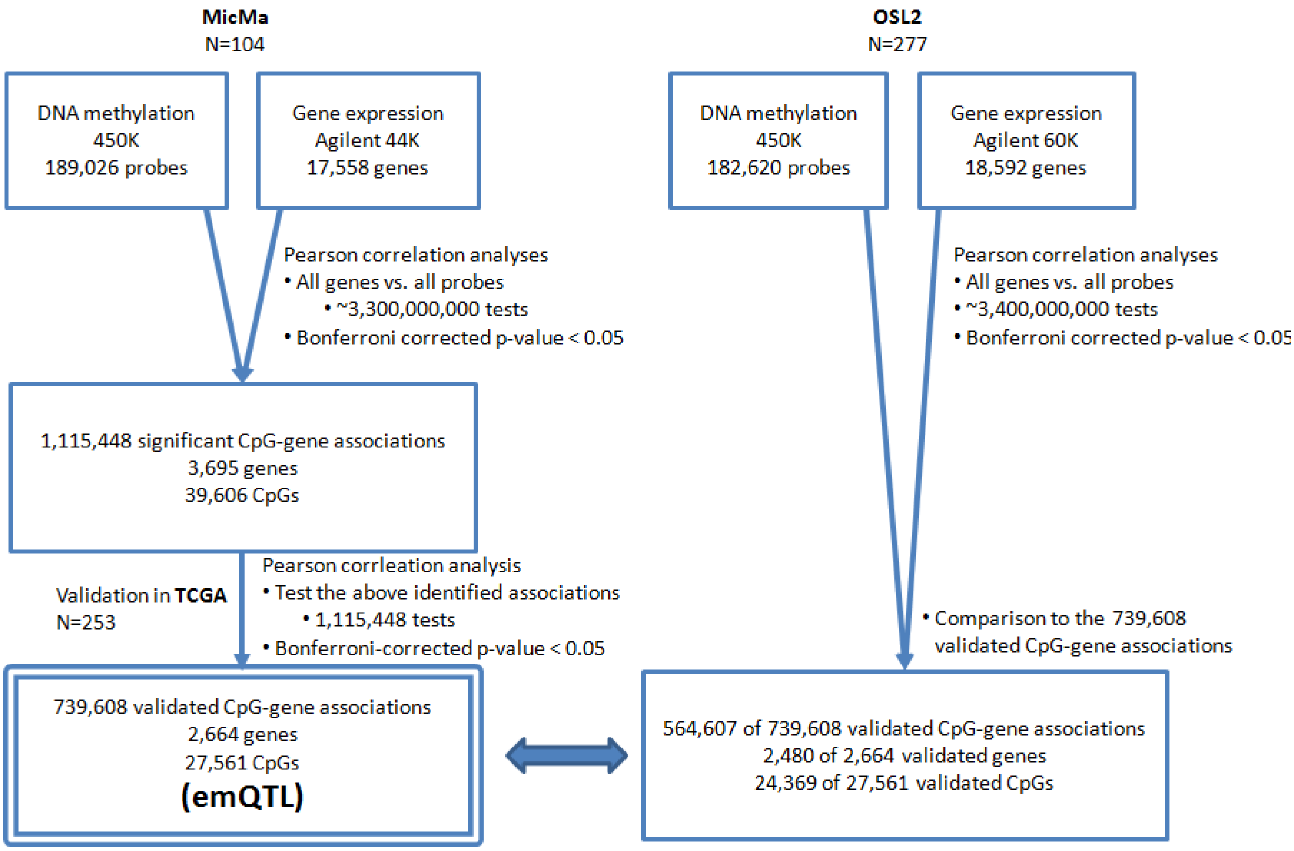

Supplementary Figure 1: **Flow chart of the identification of the significant genome-wide associations between DNA methylation and gene expression (emQTL).**

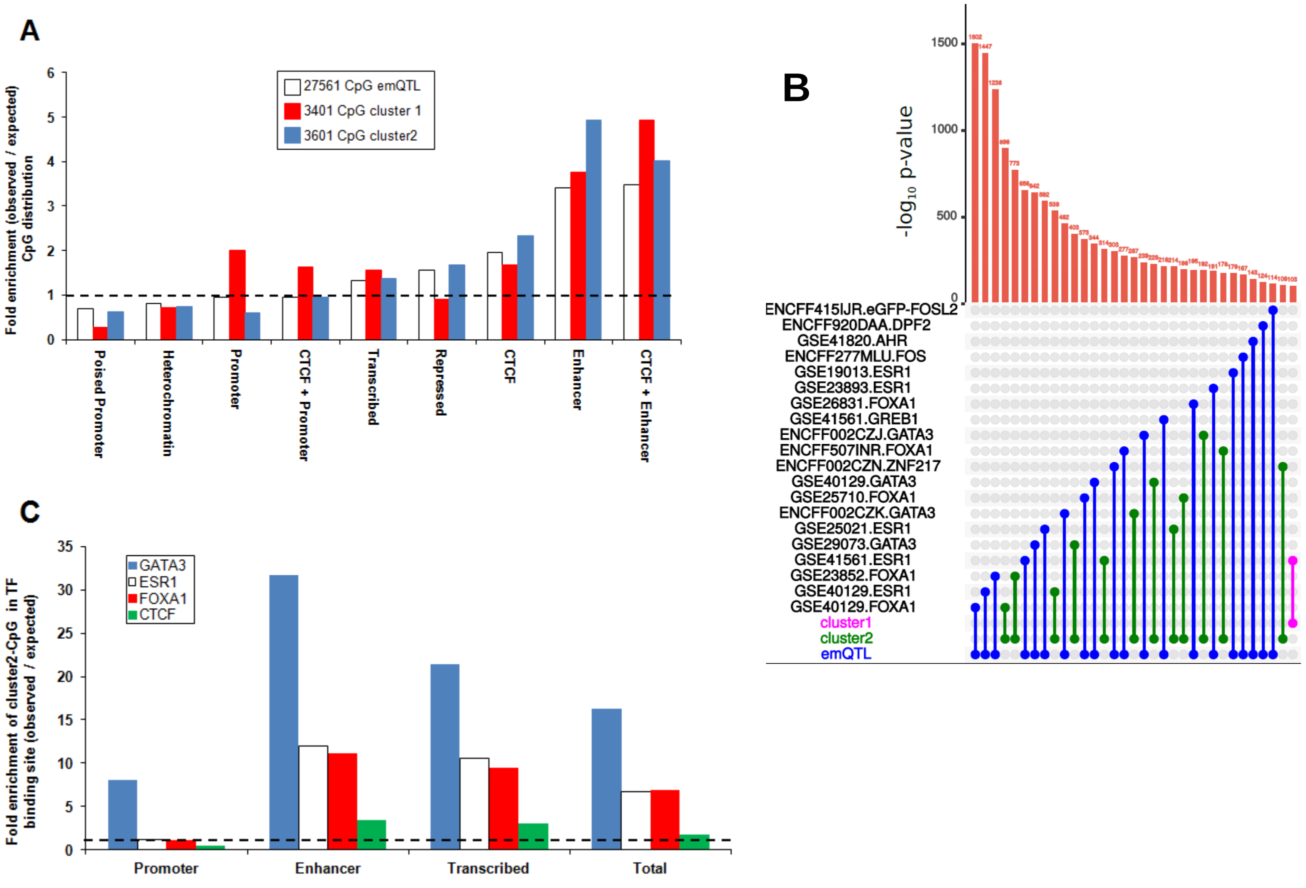

Supplementary Figure 2: Genomic location of emQTL according to ChromHMM and TF binding regions

A) Bar plot showing the association of emQTL, Cluster 1 and Cluster 2 across functional/regulatory regions of the genome as determined by MCF7 ChromHMM annotation (Taberlay et al. 2014). The height of the bars represents the level of enrichment measured as a ratio between the frequency of emQTL (white), Cluster 1 (red) and Cluster 2 (blue) CpGs overlapping a functional element over the expected frequency if such overlaps were to occur at random in the genome. **B)** Most significant enrichment of emQTL, Cluster1 and Cluster2 CpGs across 71 ENCODE and 40 GEO ChiP-seq experiments performed in the MCF7 cell line. The height of the bar plot represent –log(p-value) of the hypergeometric test assessing for enrichment of emQTL, Cluster1 or Cluster2 CpG over the distribution off Illumina HumanMethylation450k CpGs. **C)** Enrichment analysis of CpGs in Cluster 2 across ERα (white), GATA3 (blue), FOXA1 (red) and CTCF (green) binding regions as determined by ChIP-seq analysis. Enrichment is calculated at different genomic regions determined by MCF7 ChromHMM annotation. For this analysis some ChromHMM annotations were collapsed into one as follow: Enhancer=‘Enhancer’ and ‘Enhancer+CTCF’ and Promoter= ‘Promoter’, ‘Promoter+CTCF’ and ‘Poised Promoter’. The height of the bars represents the level of enrichment measured as a ratio between the frequency of Cluster 2-CpG in each ChIP-seq peak at specific regulatory region over the expected frequency if such overlaps were to occur at random in the genome. Statistically significant enrichments (p value < $1x10^{-10}$; hypergeometric test) are marked with an asterisk.

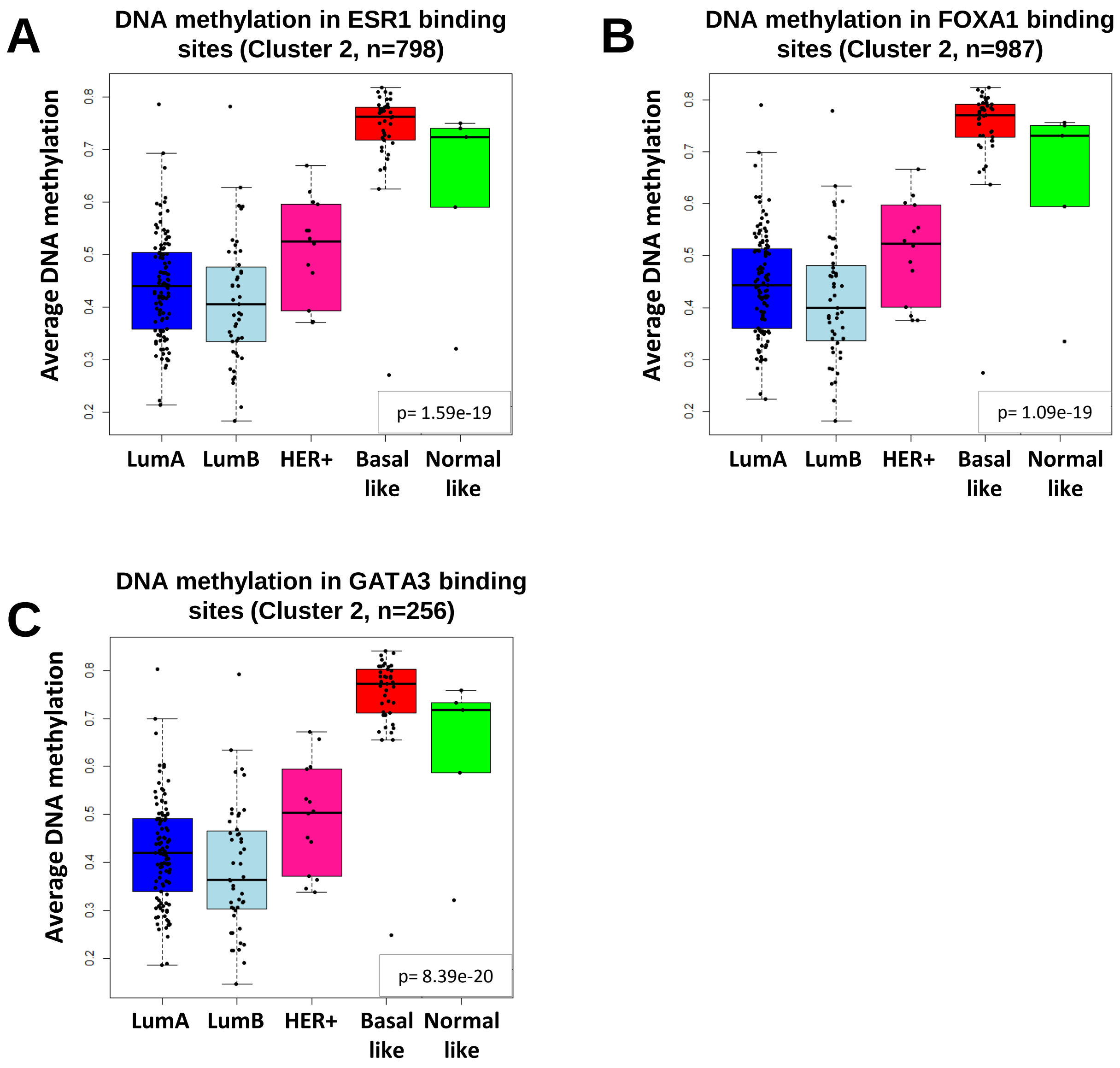

Supplementary Figure 3: Average DNA methylation of CpGs in Cluster 2 and in TF binding regions according to PAM50 subtype

Average DNA methylation of CpGs in Cluster 2 and ERα (**A**: n=798), FOXA1 (**B**: n=987) and GATA3 (**C**: n=265) binding regions defined by ChiP-seq peaks. Box plots represent the average DNA methylation of these CpGs in Luminal A (blue, n=110), Luminal B (light blue, n=48), Her2+ (pink, n=15), Basal like (red, n=42), Normal like (green, n=5) from the TCGA cohort.

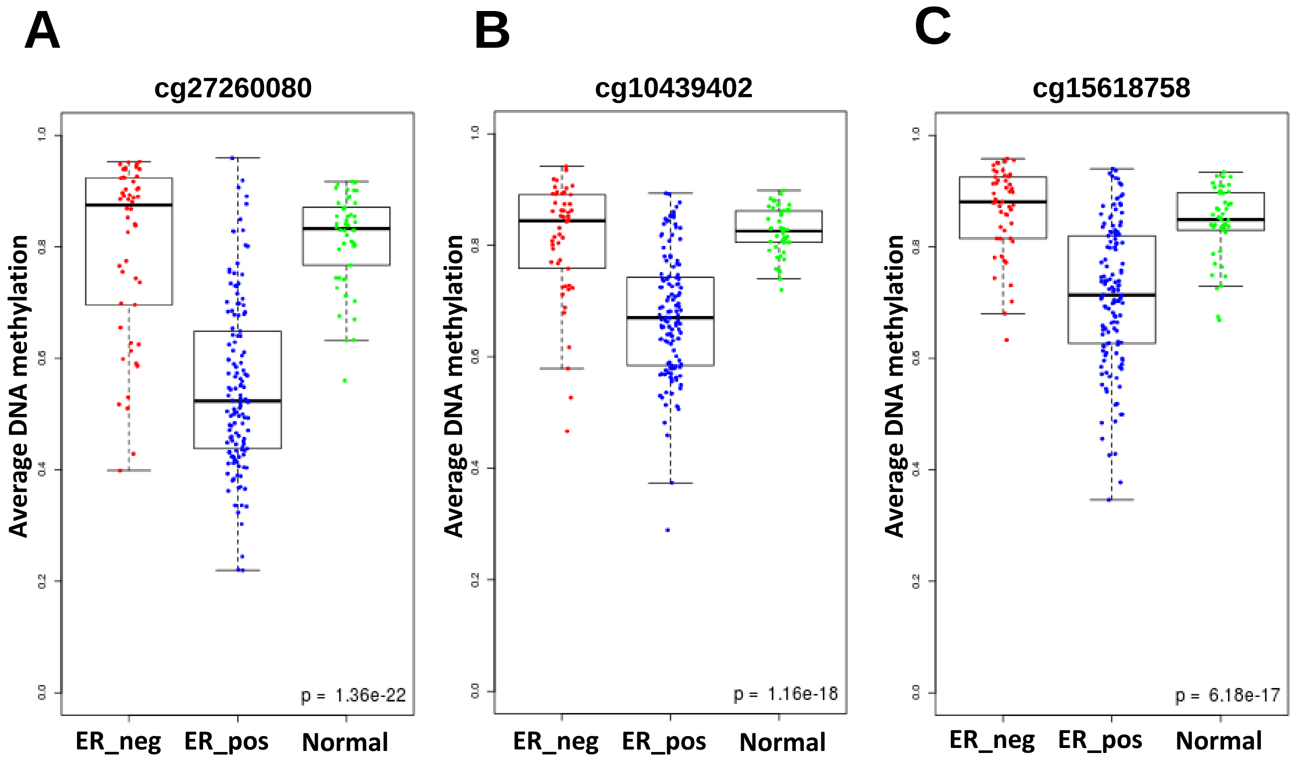

Supplementary Figure 4: Examples of CpGs at TF binding region significantly demethylated in ER-positive disease compared to healthy tissue.

The DNA methylation of three probes in Cluster 2 and TF binding regions: **A**) cg27260080, **B**) cg10439402 and **C**) cg15618758). All three probes are specifically demethylated in ER positive tumors (Kruskal Wallis p-value = $1.36x10^{-22}$, $1.16x10^{-18}$ and $6.18x10^{-17}$, respectively)

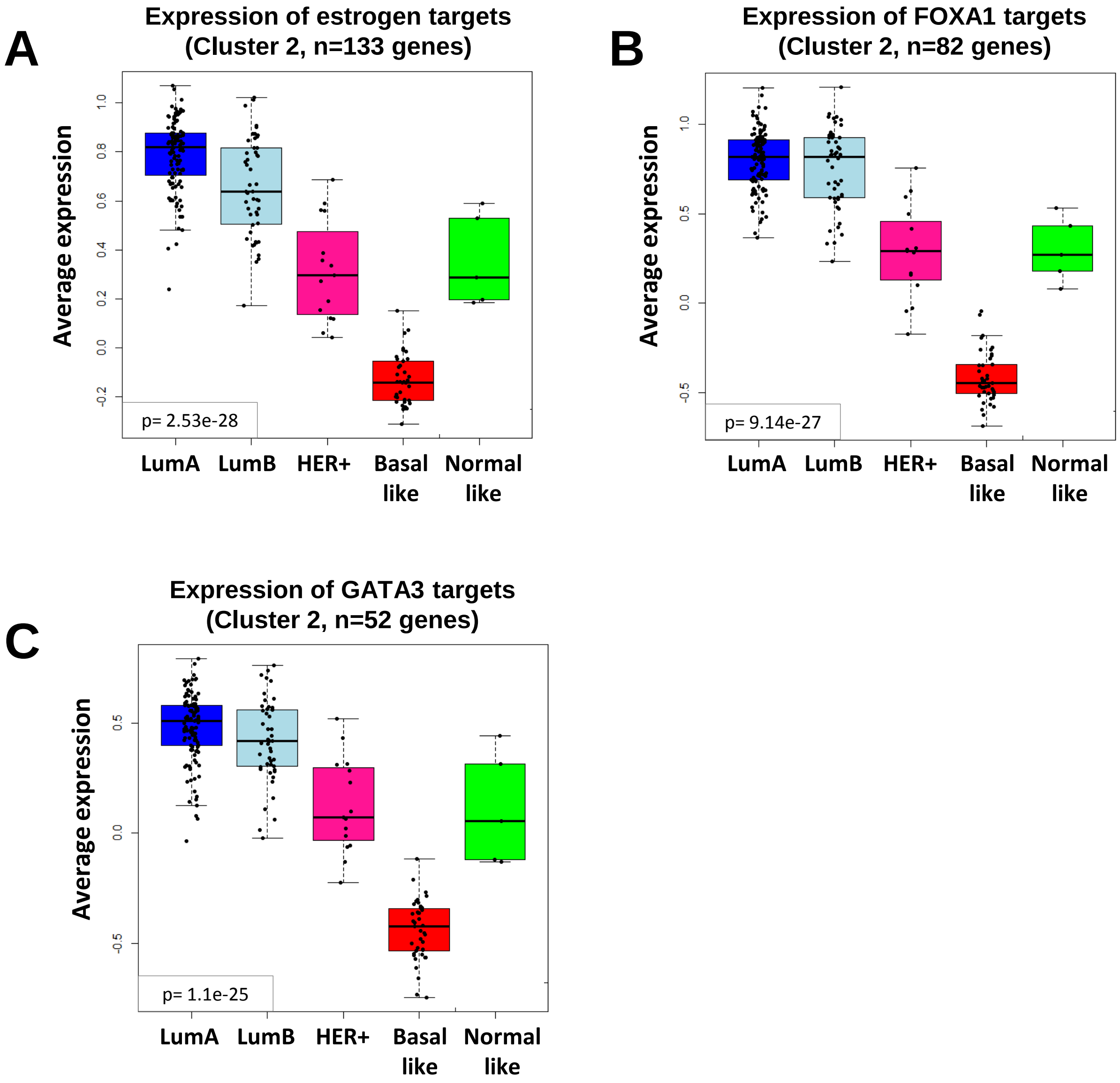

Supplementary Figure 5: Average gene expression of genes in Cluster 2 and targets TFs according to PAM50 subtype

Average expression of gene in Cluster 2 and target of ERα (**A**: n=133), FOXA1 (**B**: n=82) and GATA3 (**C**: n=52) experimentally defined by siRNA-RNAseq or Gro-seq experiments. Box plots represent the average expression of these genes in Luminal A (blue, n=110), Luminal B (light blue, n=48), Her2 enriched (pink, n=15), Basal like (red, n=42), Normal like (green, n=5) from the TCGA cohort.

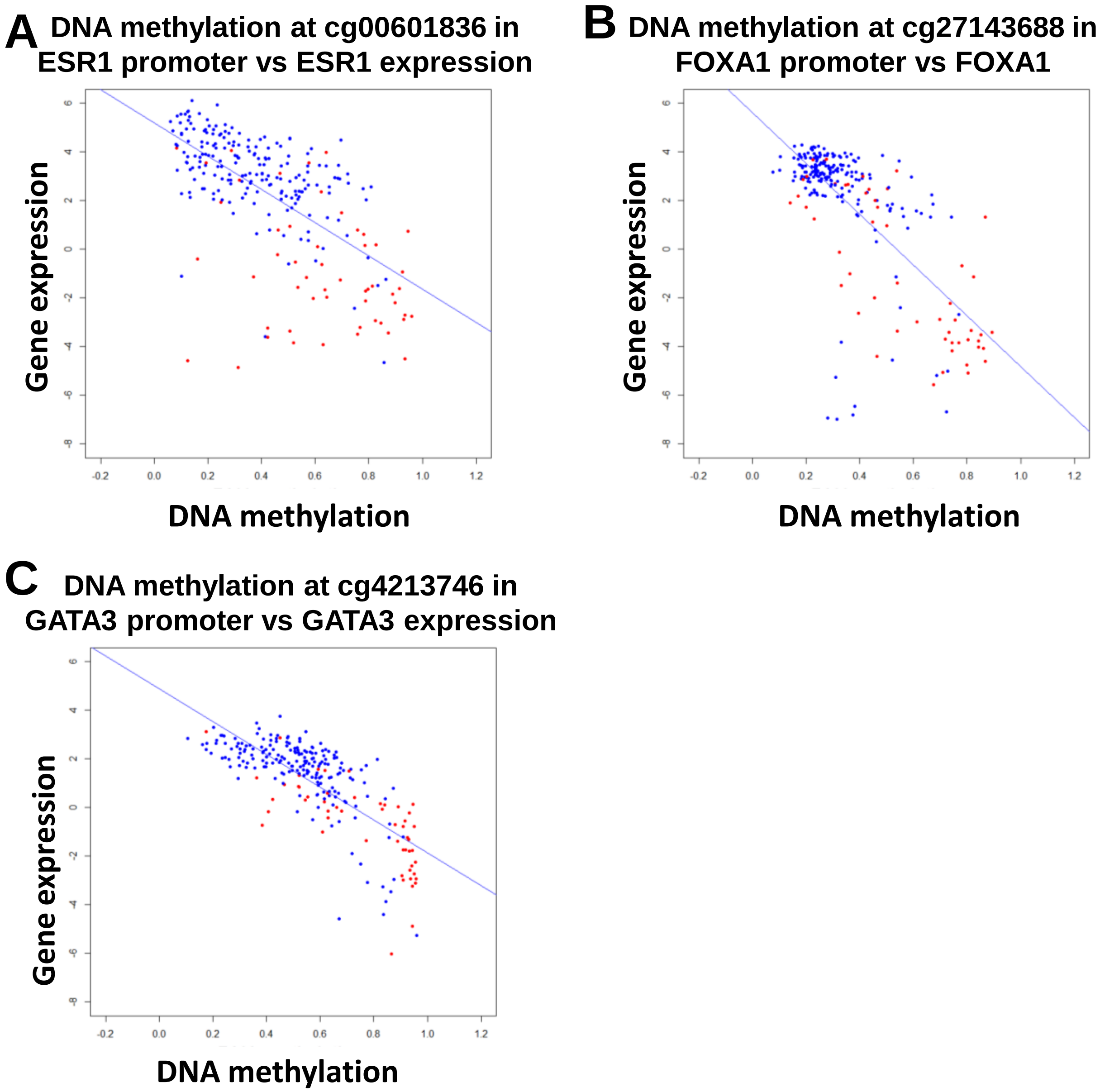

Supplementary Figure 6: Correlation between promoter methylation and expression of **A**) *ESR1*, **B**) *FOXA1* and **C**) *GATA3*.

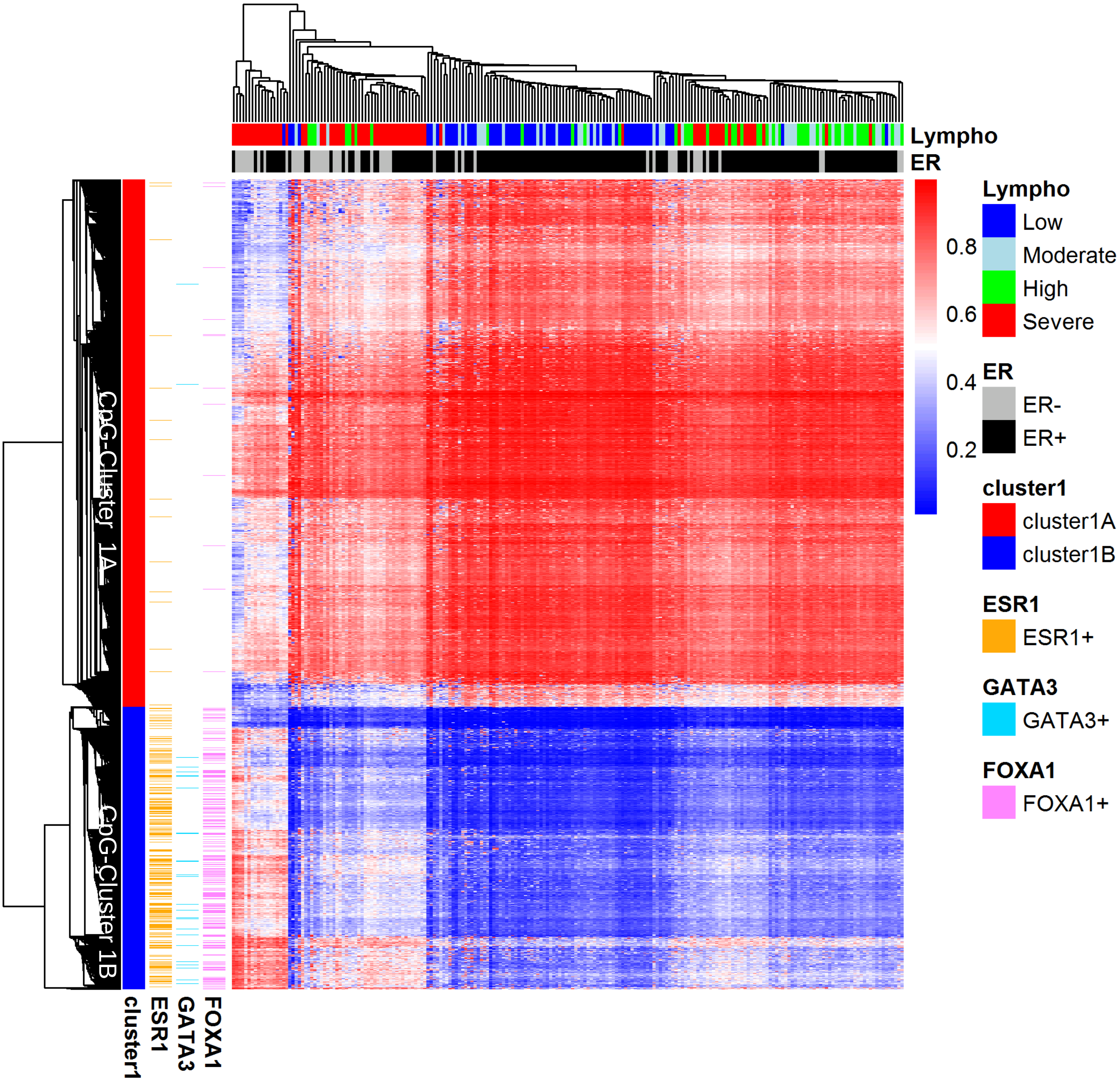

Supplementary Figure 7: Unsupervised clustering of DNA methylation in breast cancer patients using emQTL CpGs in Cluster 1.

Unsupervised clustering of DNA methylation levels of the 3401 CpGs in Cluster 1 from 213 patients of the TCGA cohort with PAM50 information. Annotation of the rows of the heatmap shows whether a CpG is located in ERα (yellow), FOXA1 (pink) or GATA3 (light blue) binding regions according to ChIP-seq experiments in MCF7 cells. Annotations of the column of the heatmap indicate histopathological features of the patients: PAM50 subtype, ER and progesterone receptor (PR) status.

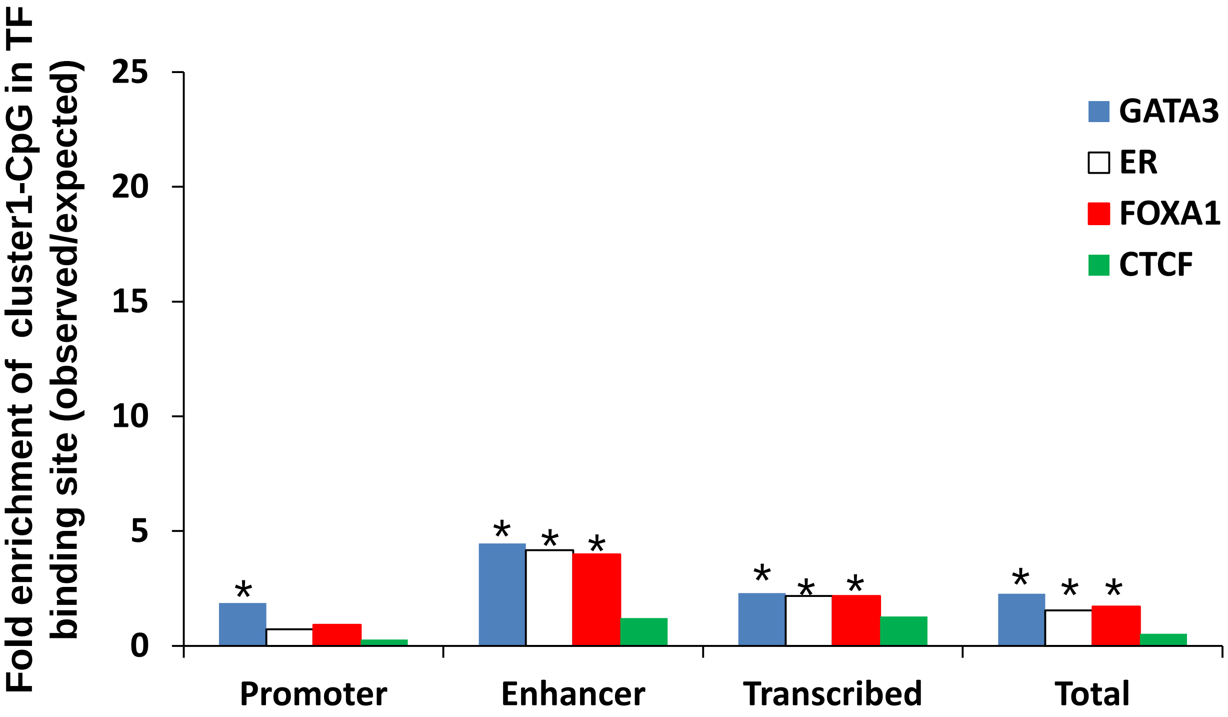

Supplementary Figure 8: Enrichment analysis of CpGs in Cluster 1 across ERα (white), GATA3 (blue), FOXA1 (red) and CTCF (green) binding regions as determined by ChIP-seq analysis. Enrichement is calculated at different genomic regions determined by MCF7 ChromHMM annotation. For this analysis some ChromHMM annotations were collapsed into one as follow: Enhancer='Enhancer' and 'Enhancer+CTCF' and Promoter= 'Promoter', 'Promoter+CTCF' and 'Poised Promoter'. The height of the bars represents the level of enrichment measured as a ratio between the frequency of Cluster 1-CpG in each ChIP-seq peak at specific regulatory region over the expected frequency if such overlaps were to occur at random in the genome. The dotted bars represent the level of enrichment of cluster2-CpGs in the respective ChIP-seq regions and ChromHMM annotations.

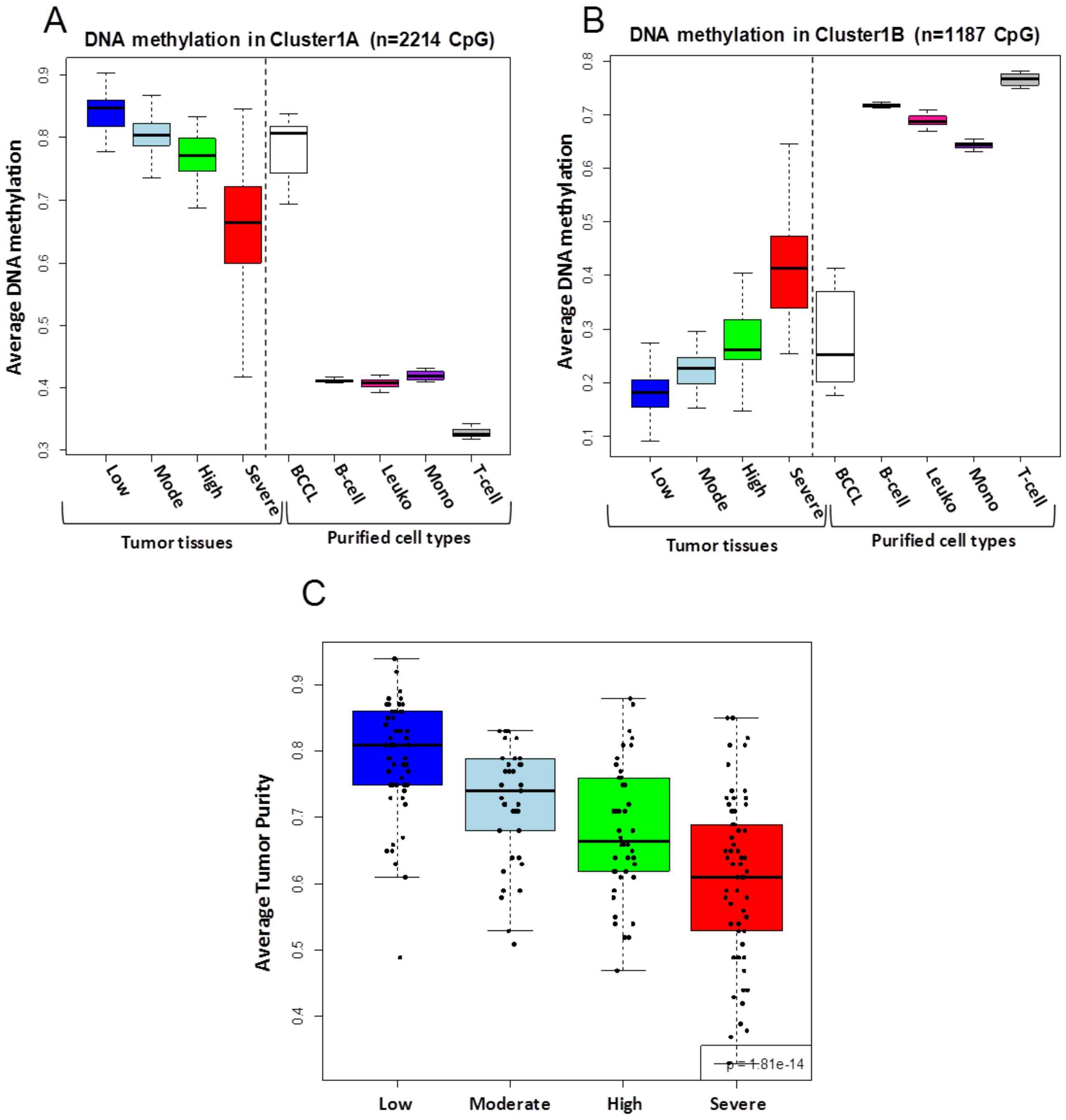

Supplementary Figure 9: DNA methylation of the 2214 CpGs of Cluster1A **(A)** or 1187 CpGs of Cluster1B **(B)** in Breast Cancer cell lines (BCCL, n=12), T cells (n=12), Leukocytes (n=12), Monocytes (n=12), B cells (n=7), TCGA tumor samples with low (n=58), moderate (n=41), high (n=46) or severe (n=69) lymphocyte infiltration. **(C)** Tumor purity measured using TCGA DNA methylation data and InfimiumPurity (Zheng et al 2017) in perspective of lymphocyte infiltration measured by Nanodissect.

Supplementary Table 1: Motif enrichment analysis ± 200bp around the CpG of Cluster 1

Motif Name	Consensus	P-value
Fli1(ETS)/CD8-FLI-ChIP-Seq(GSE20898)/Homer	NRYTTCCGGH	1,00E-12
ETS1(ETS)/Jurkat-ETS1-ChIP-Seq(GSE17954)/Homer	ACAGGAAGTG	1,00E-08
ETV1(ETS)/GIST48-ETV1-ChIP-Seq(GSE22441)/Homer	AACCGGAAGT	1,00E-08
EWS:ERG-fusion(ETS)/CADO_ES1-EWS:ERG-ChIP-Seq(SRA014231)/Homer	ATTTCCTGTN	1,00E-08
RUNX2(Runt)/PCa-RUNX2-ChIP-Seq(GSE33889)/Homer	NWAACCACADNN	1,00E-07
ETS(ETS)/Promoter/Homer	AACCGGAAGT	1,00E-07
EWS:FLI1-fusion(ETS)/SK_N_MC-EWS:FLI1-ChIP-Seq(SRA014231)/Homer	VACAGGAAAT	1,00E-07
ERG(ETS)/VCaP-ERG-ChIP-Seq(GSE14097)/Homer	ACAGGAAGTG	1,00E-06
Ets1-distal(ETS)/CD4+-PolII-ChIP-Seq(Barski et al.)/Homer	MACAGGAAGT	1,00E-06
RUNX(Runt)/HPC7-Runx1-ChIP-Seq(GSE22178)/Homer	SAAACCACAG	1,00E-05
RUNX-AML(Runt)/CD4+-PolII-ChIP-Seq(Barski et al.)/Homer	GCTGTGGTTW	1,00E-05
Elk1(ETS)/Hela-Elk1-ChIP-Seq(GSE31477)/Homer	HACTTCCGGY	1,00E-05
ELF1(ETS)/Jurkat-ELF1-ChIP-Seq(SRA014231)/Homer	AVCCGGAAGT	1,00E-05
IRF2(IRF)/Erythroblas-IRF2-ChIP-Seq(GSE36985)/Homer	GAAASYGAAASY	1,00E-04
Elk4(ETS)/Hela-Elk4-ChIP-Seq(GSE31477)/Homer	NRYTTCCGGY	1,00E-04
Nkx3.1(Homeobox)/LNCaP-Nkx3.1-ChIP-Seq(GSE28264)/Homer	AAGCACTTAA	1,00E-04
E2F7(E2F)/Hela-E2F7-ChIP-Seq(GSE32673)/Homer	VDTTTCCCGCCA	1,00E-04
PRDM1(Zf)/Hela-PRDM1-ChIP-Seq(GSE31477)/Homer	ACTTTCACTTTC	1,00E-03
GABPA(ETS)/Jurkat-GABPa-ChIP-Seq(GSE17954)/Homer	RACCGGAAGT	1,00E-03
Srebp1a(bHLH)/HepG2-Srebp1a-ChIP-Seq(GSE31477)/Homer	RTCACSCCAY	1,00E-03
IRF4(IRF)/GM12878-IRF4-ChIP-Seq(GSE32465)/Homer	ACTGAAACCA	1,00E-03
IRF1(IRF)/PBMC-IRF1-ChIP-Seq(GSE43036)/Homer	GAAAGTGAAAGT	1,00E-03
bZIP:IRF(bZIP,IRF)/Th17-BatF-ChIP-Seq(GSE39756)/Homer	NAGTTTCABTHTGACTNW	1,00E-03
RUNX1(Runt)/Jurkat-RUNX1-ChIP-Seq(GSE29180)/Homer	AAACCACARM	1,00E-03
NFkB-p50,p52(RHD)/Monocyte-p50-ChIP-Chip(Schreiber et al.)/Homer	GGGGGAATCCCC	1,00E-03
SpiB(ETS)/OCILY3-SPIB-ChIP-Seq(GSE56857)/Homer	AAAGRGGAAGTG	1,00E-02
PU.1(ETS)/ThioMac-PU.1-ChIP-Seq(GSE21512)/Homer	AGAGGAAGTG	1,00E-02
Eomes(T-box)/H9-Eomes-ChIP-Seq(GSE26097)/Homer	ATTAACACCT	1,00E-02
ISRE(IRF)/ThioMac-LPS-Expression(GSE23622)/Homer	AGTTTCASTTTC	1,00E-02
RLR1?/SacCer-Promoters/Homer	WTTTTCYYTTTT	1,00E-02
ETS:RUNX(ETS,Runt)/Jurkat-RUNX1-ChIP-Seq(GSE17954)/Homer	RCAGGATGTGGT	1,00E-02
PU.1-IRF(ETS:IRF)/Bcell-PU.1-ChIP-Seq(GSE21512)/Homer	MGGAAGTGAAAC	1,00E-02

Supplementary Table 2: Motif enrichment analysis ± 200bp around the CpG of Cluster 2

Motif Name	Consensus	P-value
FOXA1(Forkhead)/MCF7-FOXA1-ChIP-Seq(GSE26831)/Homer	WAAGTAAACA	1,00E-21
FOXA1(Forkhead)/LNCAP-FOXA1-ChIP-Seq(GSE27824)/Homer	WAAGTAAACA	1,00E-21
Foxa2(Forkhead)/Liver-Foxa2-ChIP-Seq(GSE25694)/Homer	CYTGTTTACWYW	1,00E-16
Fox:Ebox(Forkhead,bHLH)/Panc1-Foxa2-ChIP-Seq(GSE47459)/Homer	NNNVCTGWGYAAACASN	1,00E-12
GATA(Zf),IR3/iTreg-Gata3-ChIP-Seq(GSE20898)/Homer	NNNNNBAGATAWYATCTVHN	1,00E-11
GATA(Zf),IR4/iTreg-Gata3-ChIP-Seq(GSE20898)/Homer	NAGATWNBNATCTNN	1,00E-11
Gata4(Zf)/Heart-Gata4-ChIP-Seq(GSE35151)/Homer	NBWGATAAGR	1,00E-09
Gata2(Zf)/K562-GATA2-ChIP-Seq(GSE18829)/Homer	BBCTTATCTS	1,00E-07
FOXA1:AR(Forkhead,NR)/LNCAP-AR-ChIP-Seq(GSE27824)/Homer	AGTAAACAAAAAAGAACAND	1,00E-07
GATA3(Zf)/iTreg-Gata3-ChIP-Seq(GSE20898)/Homer	AGATAASR	1,00E-07
PHA-4(Forkhead)/cElegans-Embryos-PHA4-ChIP-Seq(modEncode)/Homer	KTGTTTGC	1,00E-07
Gata1(Zf)/K562-GATA1-ChIP-Seq(GSE18829)/Homer	SAGATAAGRV	1,00E-06
PQM-1(?)/cElegans-L3-ChIP-Seq(modEncode)/Homer	ACTGATAAGA	1,00E-05
ELT-3(Gata)/cElegans-L1-ELT3-ChIP-Seq(modEncode)/Homer	AWTGATAAGA	1,00E-04
Pax7(Paired,Homeobox),long/Myoblast-Pax7-ChIP-Seq(GSE25064)/Homer	TAATCHGATTAC	1,00E-04
FOXP1(Forkhead)/H9-FOXP1-ChIP-Seq(GSE31006)/Homer	NYYTGTTTACHN	1,00E-04
Lhx3(Homeobox)/Neuron-Lhx3-ChIP-Seq(GSE31456)/Homer	ADBTAATTAR	1,00E-03
GATA3(Zf),DR8/iTreg-Gata3-ChIP-Seq(GSE20898)/Homer	AGATSTNDNNDSAGATAASN	1,00E-02
Tcfcp2l1(CP2)/mES-Tcfcp2l1-ChIP-Seq(GSE11431)/Homer	NRAACCRGTTYRAACCRGYT	1,00E-02
GATA3(Zf),DR4/iTreg-Gata3-ChIP-Seq(GSE20898)/Homer	AGATGKDGAGATAAG	1,00E-02
Ap4(bHLH)/AML-Tfap4-ChIP-Seq(GSE45738)/Homer	NAHCAGCTGD	1,00E-02
LXRE(NR),DR4/RAW-LXRb.biotin-ChIP-Seq(GSE21512)/Homer	RGGTTACTANAGGTCA	1,00E-02
Rbpj1(?)/Panc1-Rbpj1-ChIP-Seq(GSE47459)/Homer	HTTTCCCASG	1,00E-02
Mef2c(MADS)/GM12878-Mef2c-ChIP-Seq(GSE32465)/Homer	DCYAAAAATAGM	1,00E-02
HIF-1a(bHLH)/MCF7-HIF1a-ChIP-Seq(GSE28352)/Homer	TACGTGCV	1,00E-02
CES-1(Homeobox)/cElegans-L1-CES1-ChIP-Seq(modEncode)/Homer	AAATTSAATTTN	1,00E-02

Supplementary Table 3: Enrichment in Transcription factor binding regions +/- 200 bp around the CpG in Cluster 1: Regions ranging +/- 200bp of CpG in Cluster 1 were overlapped with all ENCODE phase 2 ChIP-seq data using the HOMER software mergePeaks.pl function. The most significant overlap between ChIP-seq experiments are presented

Encode-ChIP-seq experiments	log p-value
wgEncodeAwgTfbsHaibGm12878Runx3sc101553V0422111UniPk.narrowPeak	-1307.191394
wgEncodeAwgTfbsHaibGm12878Ebf1sc137065Pcr1xUniPk.narrowPeak	-1032.143885
wgEncodeAwgTfbsHaibGm12878Pol2Pcr2xUniPk.narrowPeak	-905.295998
wgEncodeAwgTfbsHaibGm12878Pu1Pcr1xUniPk.narrowPeak	-756.838075
wgEncodeAwgTfbsHaibGm12891Pu1Pcr1xUniPk.narrowPeak	-719.308023
wgEncodeAwgTfbsHaibGm12878Nficsc81335V0422111UniPk.narrowPeak	-695.542770
wgEncodeAwgTfbsHaibGm12878Pol24h8Pcr1xUniPk.narrowPeak	-690.734033
wgEncodeAwgTfbsHaibGm12878Elf1sc631V0416101UniPk.narrowPeak	-676.314099
wgEncodeAwgTfbsSydhGm12891Pol2IggmusUniPk.narrowPeak	-674.196282
wgEncodeAwgTfbsSydhGm12891NfkbTnfaIggrabUniPk.narrowPeak	-669.386861
wgEncodeAwgTfbsSydhGm12892Pol2IggmusUniPk.narrowPeak	-660.914294
wgEncodeAwgTfbsHaibGm12878Pax5n19Pcr1xUniPk.narrowPeak	-651.045595
wgEncodeAwgTfbsSydhGm18505Pol2IggmusUniPk.narrowPeak	-617.459111
wgEncodeAwgTfbsHaibGm12878Tcf12Pcr1xUniPk.narrowPeak	-616.588019
wgEncodeAwgTfbsHaibGm12878BatfPcr1xUniPk.narrowPeak	-606.686300
wgEncodeAwgTfbsSydhGm12878Pol2IggmusUniPk.narrowPeak	-604.504914
wgEncodeAwgTfbsHaibGm12878Bcl11aPcr1xUniPk.narrowPeak	-590.966615
wgEncodeAwgTfbsSydhGm19193Pol2IggmusUniPk.narrowPeak	-581.292330
wgEncodeAwgTfbsSydhGm12878P300IggmusUniPk.narrowPeak	-571.425067
wgEncodeAwgTfbsSydhGm12878Ebf1sc137065UniPk.narrowPeak	-554.997897
wgEncodeAwgTfbsHaibGm12878Atf2sc81188V0422111UniPk.narrowPeak	-553.517141
wgEncodeAwgTfbsSydhGm19099Pol2IggmusUniPk.narrowPeak	-549.286773
wgEncodeAwgTfbsHaibGm12878Sp1Pcr1xUniPk.narrowPeak	-546.174185
wgEncodeAwgTfbsHaibGm12878Foxm1sc502V0422111UniPk.narrowPeak	-533.562676
wgEncodeAwgTfbsHaibGm12892Pol2V0416102UniPk.narrowPeak	-527.597142
wgEncodeAwgTfbsSydhGm12878Mxi1IggmusUniPk.narrowPeak	-514.472053
wgEncodeAwgTfbsSydhNb4MaxUniPk.narrowPeak	-513.648998
wgEncodeAwgTfbsHaibGm12878Pmlsc71910V0422111UniPk.narrowPeak	-511.995511
wgEncodeAwgTfbsHaibGm12878Tcf3Pcr1xUniPk.narrowPeak	-511.239687
wgEncodeAwgTfbsHaibGm12878Mta3sc81325V0422111UniPk.narrowPeak	-510.966613
wgEncodeAwgTfbsHaibGm12878Pou2f2Pcr1xUniPk.narrowPeak	-509.696966

Supplementary Table 4: Enrichment in Transcription factor binding regions +/- 200 bp

around the CpG in Cluster 2:: Regions ranging +/- 200bp of CpG in Cluster 1 and 2 were overlapped with all ENCODE phase 2 ChIP-seq data using the HOMER software mergePeaks.pl function. The ten most significant overlap between ChIP-seq experiments are presented with the TF ChIPped , the cell line used and the ID of the ENCODE experiment.

**Encode-ChIP-seq experiments**	**log p-value**
wgEncodeAwgTfbsHaibT47dGata3sc268V0416102Dm002p1hUniPk.narrowPeak	-440.982472
wgEncodeAwgTfbsHaibHepg2Foxa1sc6553V0416101UniPk.narrowPeak	-418.416417
wgEncodeAwgTfbsHaibT47dFoxa1sc6553V0416102Dm002p1hUniPk.narrowPeak	-395.686496
wgEncodeAwgTfbsHaibHepg2Foxa1sc101058V0416101UniPk.narrowPeak	-393.268593
wgEncodeAwgTfbsHaibHepg2Foxa2sc6554V0416101UniPk.narrowPeak	-360.501327
wgEncodeAwgTfbsSydhMcf7Gata3sc269UcdUniPk.narrowPeak	-335.628062
wgEncodeAwgTfbsHaibGm12878Runx3sc101553V0422111UniPk.narrowPeak	-312.729035
wgEncodeAwgTfbsHaibGm12878Ebf1sc137065Pcr1xUniPk.narrowPeak	-262.653251
wgEncodeAwgTfbsSydhNb4MaxUniPk.narrowPeak	-248.992820
wgEncodeAwgTfbsSydhNb4CmycUniPk.narrowPeak	-226.023248
wgEncodeAwgTfbsSydhShsy5yGata2UcdUniPk.narrowPeak	-223.782382
wgEncodeAwgTfbsHaibT47dEralphaaPcr2xGen1hUniPk.narrowPeak	-216.280272
wgEncodeAwgTfbsHaibHepg2P300V0416101UniPk.narrowPeak	-215.839981
wgEncodeAwgTfbsHaibT47dEralphaaV0416102Est10nm1hUniPk.narrowPeak	-213.533919
wgEncodeAwgTfbsSydhMcf10aesCfosTam112hHvdUniPk.narrowPeak	-198.945163
wgEncodeAwgTfbsSydhMcf7Znf217UcdUniPk.narrowPeak	-196.413545
wgEncodeAwgTfbsHaibSknshraP300V0416102UniPk.narrowPeak	-193.962428
wgEncodeAwgTfbsHaibGm12878Elf1sc631V0416101UniPk.narrowPeak	-187.934791
wgEncodeAwgTfbsUtaMcf7CmycVehUniPk.narrowPeak	-187.194725
wgEncodeAwgTfbsSydhMcf10aesCfosEtoh01HvdUniPk.narrowPeak	-183.046813
wgEncodeAwgTfbsSydhHelas3CebpbIggrabUniPk.narrowPeak	-180.557658
wgEncodeAwgTfbsHaibHepg2Fosl2V0416101UniPk.narrowPeak	-179.826868
wgEncodeAwgTfbsHaibA549Fosl2V0422111Etoh02UniPk.narrowPeak	-177.755509
wgEncodeAwgTfbsHaibHepg2Nficsc81335V0422111UniPk.narrowPeak	-175.120039
wgEncodeAwgTfbsSydhMcf10aesCfosTam14hHvdUniPk.narrowPeak	-174.195838
wgEncodeAwgTfbsSydhMcf7Tcf7l2UcdUniPk.narrowPeak	-172.792227
wgEncodeAwgTfbsHaibA549GrPcr1xDex50nmUniPk.narrowPeak	-171.773724
wgEncodeAwgTfbsHaibGm12878Nficsc81335V0422111UniPk.narrowPeak	-170.925082
wgEncodeAwgTfbsSydhImr90CebpbIggrabUniPk.narrowPeak	-170.316701
wgEncodeAwgTfbsHaibGm12891Pu1Pcr1xUniPk.narrowPeak	-170.023966
wgEncodeAwgTfbsSydhMcf10aesStat3Etoh01bUniPk.narrowPeak	-169.954835
wgEncodeAwgTfbsHaibGm12878Tcf12Pcr1xUniPk.narrowPeak	-164.352192
wgEncodeAwgTfbsHaibGm12878Pu1Pcr1xUniPk.narrowPeak	-163.737743
wgEncodeAwgTfbsSydhMcf10aesStat3Tam112hHvdUniPk.narrowPeak	-162.336937
wgEncodeAwgTfbsHaibK562MaxV0416102UniPk.narrowPeak	-162.304054
wgEncodeAwgTfbsSydhMcf10aesStat3Etoh01cUniPk.narrowPeak	-160.455133
wgEncodeAwgTfbsHaibHepg2Hdac2sc6296V0416101UniPk.narrowPeak	-157.295885
wgEncodeAwgTfbsHaibHuvecPol24h8V0416101UniPk.narrowPeak	-156.754475
wgEncodeAwgTfbsSydhGm12891NfkbTnfaIggrabUniPk.narrowPeak	-156.026894
wgEncodeAwgTfbsHaibHepg2RxraPcr1xUniPk.narrowPeak	-155.394755
wgEncodeAwgTfbsSydhMcf7Gata3UcdUniPk.narrowPeak	-155.234500
wgEncodeAwgTfbsSydhMcf10aesCmycTam14hHvdUniPk.narrowPeak	-154.414784
wgEncodeAwgTfbsHaibHepg2JundPcr1xUniPk.narrowPeak	-154.209765
wgEncodeAwgTfbsHaibGm12878Pax5n19Pcr1xUniPk.narrowPeak	-152.820983
wgEncodeAwgTfbsHaibA549Tcf12V0422111Etoh02UniPk.narrowPeak	-150.979321

Supplementary Table 5: Subtype specific emQTL analysis. Number of significant associations, CpGs or Genes is shown, and the percentage of rediscovered associations, CpGs or genes is shown in parenthesis.

	Luminal A<br>N=236	LuminalB<br>N=137	Her2 enriched<br>N=49	Basal-like<br>N=92	Normal-like<br>N=44
**Total number of associations**	4,689,557	1,647,259	21,161	2,036,164	178
**emQTL rediscovered**	244,388 (33.0%)	149,841 (20.3%)	4,212 (0.57%)	116,206 (15.7%)	17 (0.00023%)
**Cluster 1 genes rediscovered**	159 (98.8%)	159 (98.8%)	96 (59.6%)	153 (95,0%)	16 (9.9%)
**Cluster 1 CpGs rediscovered**	2,471 (72.7%)	2,070 (60.9%)	1,082 (31.8%)	2,389 (70.2%)	4 (0.12%)
**Cluster 2 genes rediscovered**	137 (50.6%)	115 (42.4%)	6 (2.2%)	14 (5.2%)	6 (2.2%)
**Cluster 2 CpGs rediscovered**	2,514 (69.8%)	1,624 (45.1%)	74 (2.1%)	107 (3%)	13 (0.36%)
